# Orchestrating symbiosis: how bacterial auxin programs soybean nodulation

**DOI:** 10.1093/plcell/koag110

**Published:** 2026-04-10

**Authors:** Ved Prakash

**Affiliations:** Assistant Features Editor, The Plant Cell, American Society of Plant Biologists; Department of Plant Pathology, The Ohio State University, Wooster, OH 44691, United States

When we consider the term *auxin*, it is natural to assume this refers to the plant hormone. But some plant colonizing microbes are also known to synthesize and secrete auxin to regulate host physiology. For example, *Bradyrhizobium elkanii* synthesizes and secretes auxin to promote nodule formation and nitrogen fixation in legumes (Fukuhara et al. 1994). Auxin-producing bacteria have been associated with larger and increased number of nodules, and higher nitrogen fixation (Camerini et al. 2008; Bellés-Sancho et al. 2022). While Rhizobia-derived auxin is known to affect auxin homeostasis and nodule development in *Glycine max* (soybean), the mechanism behind such changes remains largely unknown.

Recently, Chaofan Chen and colleagues (Chen et al. 2026) explored the regulatory network of auxins produced by rhizobia and soybean and how this regulation affects soybean nodule development. Authors genetically modified rhizobia (*Sinorhizobium fredii* HH103 strain) to enhance auxin production by incorporating the *indole-3-acetamide hydrolase* (*iaaH)* gene from *Pseudomonas syringae.* Soybean plants inoculated with engineered rhizobia developed higher density of nodules and elevated total nitrogenase activity. Interestingly, the average nodule size, nitrogen-fixation zone, and nitrogenase activity per nodule was decreased, suggesting a complex interplay between nodule number and nitrogenase activity in response to increased microbe-derived auxin.

Nodule development can be divided into 3 phases: initiation (nodule primordium development); infection (rhizobial colonization); and maturation (symbiosome expansion and N-fixation). Each requires different proteins to complete the process. Nodule primordium development is a complex signaling process that begins when auxin accumulates in the root cortical cells underlying root hairs infected with rhizobia and is mediated through the upregulation of plant *YUCCA* genes. The authors reported increased transcription of *GmYUC2a* in nodule primordia when inoculated with the engineered rhizobia, highlighting the role of microbe-derived auxin in plant auxin homeostasis. To understand if the spatial regulation of *GmYUC2a* was important in nodule development, the authors used cell-layer-specific promoters to overexpress *GmYUC2a* in different cell layers such as epidermis, cortex, endodermis, phloem, and nitrogen-fixation zone. Surprisingly, they found that *GmYUC2a* overexpression had no impact on nodule development, except when it was overexpressed in the cortex layer. By generating stable transgenic lines overexpressing *GmYUC2a* in the cortex, the team found that the higher *GmYUC2a* expression was linked with smaller nodule size and reduced rhizobia colonization. However, the transgenic plant developed increased nodule primordium density and increased expression of several symbiosis perception genes compared with the wild-type plant, indicating that *GmYUC2a*-mediated auxin accumulation in the cortex enhances nodule primordium density, but inhibits rhizobial colonization.

Using a yeast one-hybrid screen to identify regulators of *GmYUC2a* expression, the authors identified 2 transcription factors, NF-YA9 and LBD41, which bind to the *GmYUC2a* promoter to activate and repress its expression, respectively. Interestingly, engineered rhizobia significantly induced the expression of both transcription factors in nodule primordia, but in the mature nodule, only *LBD41* expression was induced while *NT-YA9* was unaltered. Stable transgenics lines overexpressing *NF-YA9* possessed a higher nodule primordium density, but lower total nodule density, while *LBD41*-overexpressing plants exhibited reduced density of both nodule primordia and mature nodules. These findings suggested that NF-YA9 initiates *GmYUC2a* transcription early for nodule primordium initiation, while LBD41 later acts as a negative regulator of *GmYUC2a* expression to facilitate rhizobial colonization. One of the crucial findings from this research was that the symbiosis transcription factor, NIN1a, also binds to the promoter of *GmYUC2a* in symbiotic cells and activates its transcription, thereby promoting symbiosome expansion.

Chen and colleagues demonstrated that rhizobia-derived auxin enhances plant auxin accumulation by activating the *GmYUC2a* gene and characterized the roles of 3 transcription factors which spatiotemporally regulate its expression. The coordinated regulation of auxin homeostasis therefore controls primordium development via high auxin accumulation, rhizobial colonization by restricting auxin levels, and symbiosome expansion by again increasing auxin accumulation (Fig. 1). We know that pathogens have evolved to control host physiology, and the same is certainly true for beneficial microbes. Future questions would include uncovering what other host-manipulation strategies did beneficial microbes evolve? Did the beneficial microbes acquire the ability to produce auxin primarily to manipulate host pathways? While this study explored how variation in the spaciotemporal distribution and regulation of auxin coordinates nodule development, how other plant hormones affect this process would also be interesting to follow up on. These findings offer a blueprint for engineering more efficient nitrogen-fixing crops, potentially reducing the global agricultural reliance on synthetic fertilizers.

**Figure 1:**
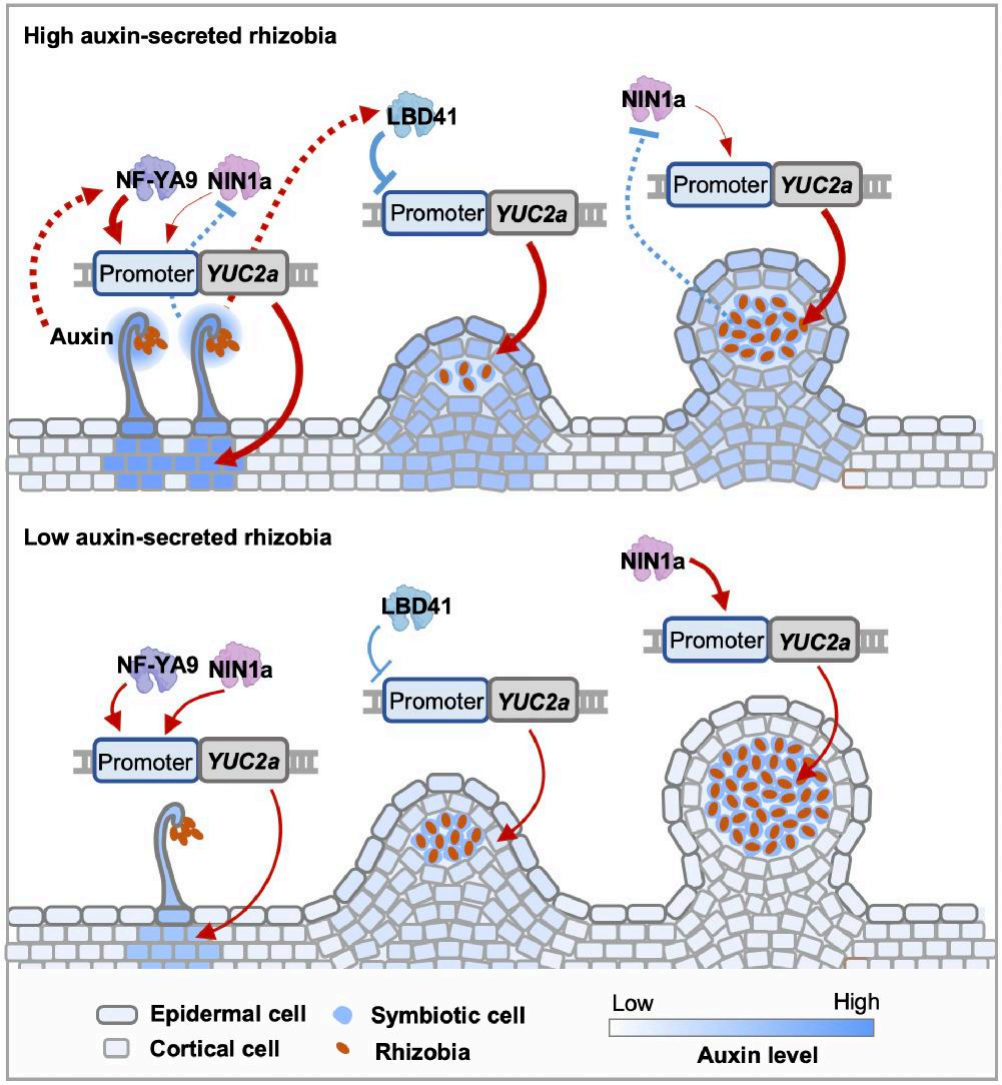
Proposed model describing the role of high and low auxin secreting rhizobia in coordinating the expression of soybean genes involved in nodule development. In the initial phase, rhizobia-derived auxin activates *NF-YA9* to initiate *YUC2a* expression and nodule primordium initiation. In the middle phase, rhizobia-derived auxin promotes *LBD41* expression which inhibits *YUC2a* expression to promote rhizobial colonization. In the late phase, NIN1a activates *YUC2a*, enhancing plant auxin level and nodule maturation. Reprinted from Chen et al. (2026), Figure 10.

## Recent related articles in *The Plant Cell:*

- Wang et al. (2025) provided genetic evidence showing that legume glutamyl-tRNA reductase-2 (GluTR2) is the primary driver of heme biosynthesis in nodule to provide the necessary heme for leghemoglobin and precursors for the nitrogen-fixing rhizobia.
- Lee et al. (2024) identified that the evolutionarily conserved protein EARLY NODULIN93 (ENOD93) acts as a subunit of mitochondrial cytochrome *c* oxidase (Complex IV) and regulates the efficiency of ATP production. This supports the high energy demands of nitrogen-linked processes and root growth.
- Yu et al. (2024) identified that *NODULES WITH ACTIVATED DEFENSE1* (*NAD1*) is present only in nitrogen-fixing clade plants and was specifically induced in nodules.

## Data Availability

No new data were generated or analyzed in support of this research.
